# The aging endothelium

**DOI:** 10.1530/VB-20-0013

**Published:** 2021-01-12

**Authors:** Ka Ka Ting, Paul Coleman, Yang Zhao, Mathew A Vadas, Jennifer R Gamble

**Affiliations:** 1 Centre for the Endothelium Vascular Biology Program Centenary Institute, The University of Sydney, Sydney, New South Wales, Australia

**Keywords:** endothelium, aging, age-related disease, senescence, vascular dysfunction

## Abstract

Cellular senescence is now recognized as one of the hallmarks of aging. Herein, we examine current findings on senescence of the vascular endothelium and its impacts on age-related vascular diseases. Endothelial senescence can result in systemic metabolic changes, implicating senescence in chronic diseases such as diabetes, obesity and atherosclerosis. Senolytics, drugs that eliminate senescent cells, afford new therapeutic strategies for control of these chronic diseases.

## Introduction

The endothelial cell (EC) monolayer forms the inner cellular lining of all blood vessels forming a critical interface between blood and tissue (1). Vascular endothelium is involved in physiological functions, which include regulation of blood fluidity, hemostasis and clotting, vascular tone, immune responses, inflammation, angiogenesis, and metabolism (2).

Dysfunction of the endothelium is a major contributor to cardiovascular diseases (CVD) such as stroke, atherosclerosis, hypertension and diabetes (see review by Hadi *et al*. (3)) and more recently has been shown to play a role in the severe response to COVID-19 (4). Chronological aging is the dominant risk factor for CVD, cancer and neurodegenerative diseases (5) and indeed endothelial dysfunctions including arterial stiffening (6), impaired neovascularization (7) and loss of tissue-barrier function are evident in age-related diseases (8). This review will focus on cellular aging or senescence of the vascular endothelium.

## Cellular senescence

Aging is defined as a gradual decline in organism function and is underlined by cellular aging. Current biological hallmarks of cellular aging include increased cellular/oxidative stress, DNA damage, telomere shortening, stem cell depletion, mitochondria dysfunction, epigenetics and ncRNA dysregulation, loss of proteostasis and cellular senescence (9). Cellular senescence is characterized by permanent cell cycle arrest and distinct changes in cell morphology, metabolism, chromatin reorganization, gene profiles and activation of a proinflammatory secretome, termed the senescence associated secretory phenotype, SASP (10, 11, 12). The SASP can include cytokines (e.g. IL-1α/β, IL-6), chemokines (e.g. CXCLs, CCLs), growth factors (e.g. VEGF, FGF), proteases (e.g. MMPs, TIMPs) and lipids (13). Different aging pathways can induce the senescence state but the SASP phenotype will be cell type and stimulant specific (14, 15). The SASP can maintain the senescence phenotype in the cells, induce senescence in neighboring cells and influence the inflammatory state of the microenvironment (13). The SASP is also essential for activating a specific inflammatory profile that is responsible for the removal of the senescence cells (16, 17).

Senescence was originally described as a potent mechanism, together with apoptosis, for controlling cell proliferation and malignant transformation. Now it is known to contribute to development (18). During embryonic development, cellular senescence is induced by cell fusion to form the outer layer of the placenta and contributes to the normal functioning placenta (19). Further, senescence contributes to pathogenic conditions such as liver fibrosis, (17) yet is essential for tissue homeostasis (20) and wound healing (21). In adults, senescence is a response to stress, triggered to halt the increase in potentially dysfunctional cells. However, the accumulation of senescent cells with age contributes to age-related-pathologies as is seen for example in renal dysfunction (22) and CVD (23). The clearance of senescent cells is immune-mediated and the increase in senescent cells with age maybe partially attributed to the aging immune system or immunosenescence (24, 25). In addition, senescent cells have the ability to evade the immune system by altering the expression of major histocompatibility complex (MHC) HLA-E (26), a key recognition signal required by natural killer cells and differentiated T-cells to clear senescent cells.

### Pathways of senescence

Broadly speaking, senescence can be divided into telomere-dependent replicative senescence (RS) or Hayflick’s limit (27) and stress-induced premature senescence (SIPS) (28). *In vitro*, replicative senescence can be induced through continual passaging of cells, as some cells (e.g. fibroblasts, ECs, immune cells) have a division lifespan. When the division capacity of these cells has been exhausted, based on their telomere shortening, they enter growth arrest and become senescent. There is substantial evidence that RS plays an important physiological role in tumor suppression (29).

Unlike RS, SIPS is considered to be a telomere shortening-independent process and is a rapid response characterized by random DNA damage in the genome followed by activation of the DNA damage response (DDR). SIPS can be induced by for example, oncogenic stress (30), metabolic stress (31), inflammation (32), and oxidative stress (33).

**Figure 1:**
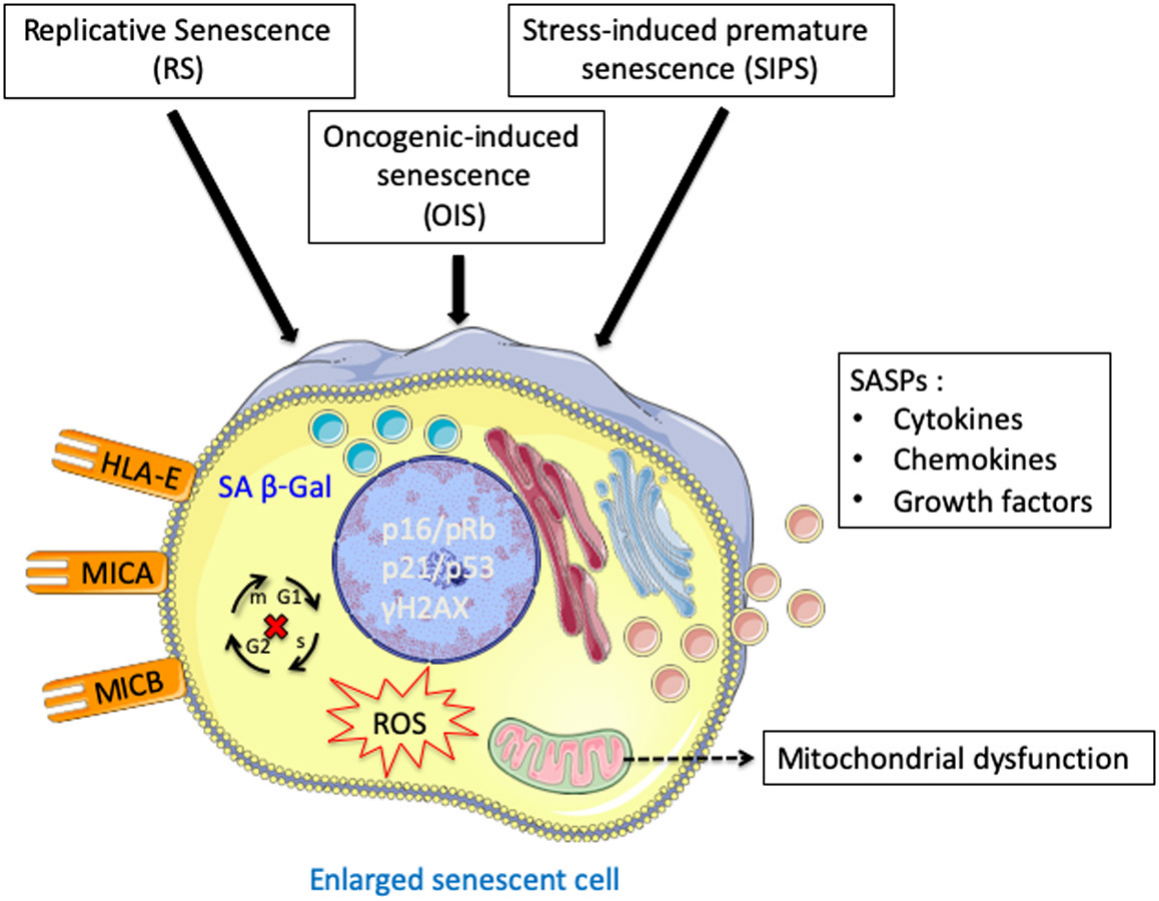
A typical senescent phenotype that is shared between different cell types and activation senescent pathways.

Most senescence is mediated through activation of the p21/p53 and p16/retinoblastoma (RB) protein tumor suppressor pathways (Fig. 1) (34). Activation of the DDR pathway and telomere dysfunction commonly induces p21/p53 dependent senescence while other stresses/stimuli are more often associated with the p16/RB pathway (34). The preference toward one pathway vs another appears to be cell type-specific (34, 35), with variation across species (36) and also stimulant dependent. For example, telomere dysfunction can lead to activation of the p53 or p16/RB pathway in human cells, but will only trigger the p53 pathway in rodent cells (36).

### Senescence markers

There is no single marker that defines a senescent cell (37) and this has hindered the field. A panel of markers in combination with either p21, p16 or p53 is used to denote senescence (Table 1).

**Table 1:** The different markers of senescence that are commonly used.

Senescence marker	Description
SA β–Gal	Senescence-associated beta-galactosidase is an enzymatic stain that can be used on cells and tissues (Fig. 1) (38), with its activity associated with an increase in lysosomes biogenesis found in senescent cells (39).
γH2AX	Histone H2A variant is DDR marker that is phosphorylated at serine 139 upon DNA double-strand breaks (40).
	γH2AX can also be used to detect nuclear membrane bleb structures that contain cytosolic chromatin fragments (CCFs) in the cytoplasmic compartment of senescent cells (41, 42).
	CCFs are known to be involved in the secretion of proinflammatory SASP through the activation of the cGAS-STING-NFκB pathway (42).
	Recently, it has been shown that impairment of autophagy is linked to CCFs formation, and metformin-induced activation of autophagy can reduce CCFs levels and suppress SASP secretion (43).
	CCFs and SASP profile in senescent primary human fibroblasts have also been associated with mitochondrial dysfunction via a retrograde ROS-JNK signaling pathway (44).
Lamin B1	Is a protein of the nuclear envelope and its expression is reduced during SIPS-associated nuclear membrane blebbing (45, 46).
Ki67, BrdU, PCNA	Are typical proliferation markers used to detect growth arrest (47, 48). High expression of these markers correlates with a high proliferation rate, and their decrease in expression is used in conjunction with other markers to confirm cellular senescence.

### Pathways that regulate senescence/SASP

The SASP is driven largely by proinflammatory pathways involving NFκB, mTOR and p38/MAPK (38, 39, 40) often activated through the metabolic state of the cell or through paracrine effects by surrounding senescent cells.

At least four interdependent nutrient-sensing pathways act in the induction of senescence. First, NAD^+^/NADH pathway that involves NAD regulated AMPK (5’ AMP-activated protein kinase) is upstream of the p38/MAPK-NFκB axis and also impacts on the proinflammatory SASP in an independent senescence growth arrest pathway (41). In contrast, pharmacological inhibition of cluster of differentiation 38 (CD38), a nicotinamide nucleotidase (NADase), was shown to reverse the age‐related decline in NAD^+^ levels in muscle and liver, and reduce telomere‐associated DNA damage in mice. Interestingly, the SASP secretome was shown to induce CD38 expression and increase CD38‐NADase activity in non‐senescent cells, leading to the suggestion that during aging the SASP may contribute to NAD^+^ decline by upregulation of CD38 (42). Although these studies had conflicting results on the role of NAD^+^ on inflammation, it shows that NAD^+^ is critical for SASP regulation and the differences may be due to the differences in cell type and/or the type of senescence induction.

Secondly, the dysfunction of mitochondria as a major energy and reactive oxygen producers, has recently been linked to the regulation of cellular senescence and SASP, mediated through AMPK (43). However, such mitochondrial dysfunction can lead to both a proinflammatory SASP as well as a low inflammatory senescent phenotype (termed mitochondrial dysfunction-associated senescence, MiDAS) driven through AMPK-mediated p53 activation (43) and with a lowered NAD^+^/NADH ratio.

Thirdly, mTOR another known energy sensor, regulates mitochondrial homeostasis and negatively regulates autophagy. Inhibition of mTOR with rapamycin has been shown to suppress SASP through the MAPKAPK2 pathway (44).

Fourthly, the energy sensor SIRT1, belongs to a family of histone and protein deacetylases, and is positively associated with longevity through calorie restriction (45). In the heart, the deficiency of SIRT1 has been linked to oxidative stress and inflammation, with senescence of ECs and VSMCs mediated through the autophagy pathway. Overexpression of SIRT1 protected against ischemia-reperfusion injury in the heart through increased autophagy, NOS, FOXO3 activation, and deacetylation of NFκB (46).

Lastly, miRNA are upstream regulators of senescence. MiRs such as miR-217 and miR-34a increases EC senescence, through SIRT1 downregulation. Indeed, there are endothelial miRs that can also regulate SASP such as miR-155 and miR-21 (see review by Yamakuchi and Hashiguchi (47)). As most of these endothelial miRs were identified using *in vitro* studies, more* in vivo* studies are required to delineate their roles in age-related diseases.

## Endothelial cell senescence in age-related diseases

**Figure 2:**
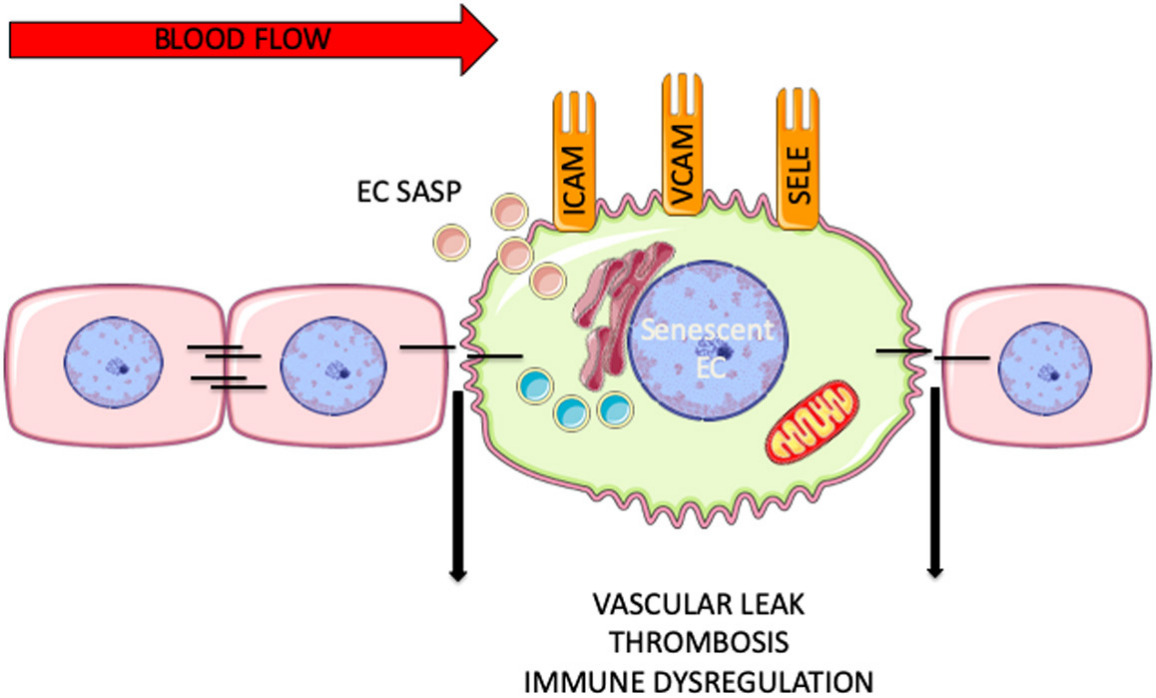
General scheme depicting the structural and functional changes in senescent ECs. Black lines represent tight or adhesion junction interactions between the ECs.

**Figure 3:**
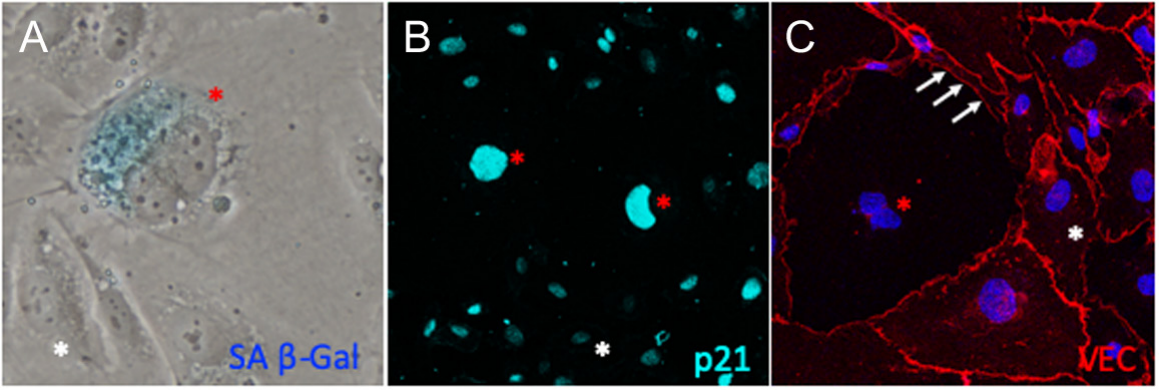
Examples of H_2_O_2_-induced senescent phenotype in human umbilical endothelial cells (HUVECs) from our lab. Senescent HUVECs depicted by red asterisk had upregulated (A) SA b-Gal, (B) nuclear p21 expression and reduced (C) VE-cadherin compared to non-senescent ECs (white asterisk).

CVD and cerebrovascular diseases are the leading causes of death in the elderly population (48). EC dysfunction is a well-accepted hallmark of age-related vascular dysfunction, with the initiation of abnormal inflammatory and thrombotic circuits, arterial stiffening and oxidative stress being central to its biology. Importantly, for our understanding of vascular aging, senescent EC accumulate in aging tissues and contribute to tissue dysfunction (49, 50, 51). Structural and functional changes in senescent ECs are summarized in Figs 2 and 3.

*In vitro* studies defining senescent ECs have demonstrated that senescence can be induced by many of the stimuli associated with aging, such as hypoxia, disturbed flow and oxidative stress (52), high glucose conditions (53), β-amyloid peptides (54) and chronic inflammatory conditions (55). These senescent ECs generate a proinflammatory SASP similar to all other cell types. The senescence is induced through the classic senescence signaling pathways, p21/p53 and p16/RB. However, ECs can also express a non-activated, potentially anti-inflammatory senescent population, as we have previously described (56), which is also induced by age-related stress (52) and by overexpression of the vascular protective gene *ARHGAP18* (a.k.a. *SENEX*) (56). This anti-inflammatory senescent phenotype is mediated through caveolae and inhibition of NFκB (57). The presence of the proinflammatory (defined as E1) and anti-inflammatory (defined as E2) senescent ECs suggests a duality of senescence in the vasculature-to both promote and limit inflammation. Confirmation of the existence *in vivo* of these cell types awaits identification of selective markers, which will allow investigations into their role in initiation and progression of disease.

Senescent ECs have been identified in many age-associated diseases. The following have been most widely studied.

### Atherosclerosis

Atherosclerosis is considered a chronic inflammatory disease, characterized by enhanced leucocyte recruitment into the lesions. Further, endothelial dysfunction is a well-known initiating event in atherogenesis (58). Plaque accumulation in the vascular system is seen from the aorta to coronary arteries (59). These plaque formations usually begin with the deposition of small cholesterol crystals in the intima and the underlying smooth muscle layer. At late stages, these plaques in the arteries can lead to clot formation and thrombosis, which will result in obstruction of blood flow and even plaque rupture.

Senescent foamy macrophages have damaging roles during the onset of atherosclerosis in mice (60). Almost two decades ago, senescent ECs were described in atherosclerotic lesions from autopsied patients (23). SA-β-gal positive ECs found at the atherosclerotic lesions were enlarged and flattened in comparison to those at non-lesional areas. Telomere lengths in aortic ECs decrease as a function of donor age and have been observed in patients with atherosclerosis (61, 62). Aortic ECs isolated from aged mice were reported to have a flat and enlarged morphology (63) and human aortic ECs induced into senescence showed EC dysfunction with enhanced proinflammatory ICAM-1 expression and reduced eNOS activity (23). Further, a higher fraction of ECs from patients with abdominal aortic aneurysm (AAA), another clinical manifestation of atherosclerosis, were positive for Ki67 demonstrating a higher proliferation rate compared to control patients (64), a likely harbinger of telomere-dependent replicative senescence.

In the context of atherosclerosis, there are a number of likely stimuli for senescence induction of the endothelium. The areas of hemodynamic stress or disturbed blood flow such as arterial bifurcations and curved areas such as the iliac and thoracic artery, and the aortic arch, are areas of atherosclerosis development. ECs in these areas are senescent, induced via the p53/p21 pathway (65). Our lab has demonstrated that disturbed flow mimicking these atherosclerotic regions, induces EC senescence (52). It is proposed that the proinflammatory SASP produced by the senescent ECs will lead to a chronic sterile inflammatory environment with vascular remodeling. Indeed, SASP cytokines such as MCP-1, PDGFB, TNFα and IL-6 are atherogenic (66) and can increase monocyte infiltration, EC turnover and smooth muscle cell movement into the sub-intimal space (67).

Hyperlipidemia and lipid oxidation are high-risk factors for atherosclerosis (68). Hypercholesterolemia is associated with increased blood levels of oxidized low-density lipoprotein (ox-LDL), well known to be involved in EC dysfunction and which induces EC senescence (69, 70). Interestingly, the protective high-density lipoproteins (HDL), which are also anti-inflammatory (71) can inhibit senescence induction (Powter EE, Rye K-A and Gamble JR, unpublished results). Some risk factors that can increase ox-LDL levels include high-fat diet, smoking, diabetes and stress (72). Many of these risk factors also correspond with an increase in free radicals, which can affect NO bioavailability, damage the endothelium and induce EC permeability. Such changes cause accumulation of LDL in the sub-endothelial space resulting in enhanced EC expression of adhesion molecules such as vascular cell adhesion molecule 1 (VCAM1), E- and P-Selectins to promote leukocyte recruitment (73). CD9, a novel senescence-related marker was reported to be increased in aged human arteries and mice (74). In mouse models of atherosclerosis, CD9 expression was co-expressed with SA-β-gal staining and was found the be upregulated in ECs and macrophages within lesions. Genetic deletion and antibody treatment against CD9 further showed CD9 is critical for inducing EC senescence and exacerbating atherosclerotic lesions. Another study reported angiopoietin-like 2 is increased in senescent aortic endothelium found in atherosclerosis mouse model and knockdown of this protein* in vivo* reduced plaque progression (75).

Beyond regulating blood pressure, angiotensin II (Ang II), a principal effector of the renin-angiotensin system (RAS), is an important signaling molecule involved in atherogenic stimuli, such as induction of oxidative stress, secretion of proinflammatory cytokines and adhesion molecules within the vessel wall (76). Ang II can induce EC senescence and dysfunction, which in turns impairs NO vasoactive function and hemodynamic stress response (77). Concurrently, the levels of angiotensin-converting enzyme (ACE) and ET-1 levels are increased (78). Clinical and experimental animal studies of ACE inhibitors have shown these prevent the progression of atherosclerosis (79, 80). Valsartan, a well-known AngII receptor blocker, has been shown to attenuate AngII-induced EC senescence and inflammation (77).

Multiple studies have shown that disturbed blood flow (81), LDL (82) and AngII (83) increase the pathogenesis of atherosclerosis by affecting Notch signaling. The Notch pathway is important in vascular development and vascular modeling (84). Ligand activation of this pathway results in the cleavage of transmembrane Notch receptor protein into intracellular and extracellular parts, whereby the intracellular domain translocates into the nucleus for transcriptional activity (84). One study showed basic-helix-loop-helix transcriptional repressors Hey1 and Hes1, which are targets of Notch activation, were increased in senescent luminal ECs within atherosclerotic plaques in ApoE^−/−^ mouse model and patients with coronary artery disease (85). The authors found that enhanced Notch1 activation resulted in telomere-dependent senescence. However, enhanced Notch1 decreased senescence in HUVECs via telomere-independent pathway (86). Further, constitutive activation of Notch pathway via increased expression of Notch1 intracellular domain was shown to induce premature senescence through p16 signaling. Apart from the usual Notch ligands (e.g. Delta, Serrate, LAG-2 family), other atherogenic proteins such as thrombospondins 1/2 and periostin, to name a few, have been shown to modulate Notch signaling (see review by Wang (87)). Both proteins are extracellular matrix proteins, and affect vascular processes like angiogenesis. Thrombospondin-1 is associated with endothelial senescence (88), but evidence of periostin and EC senescence is lacking.

### Obesity

Studies in both human and mice demonstrate physiological aging can drive senescence in adipocytes and/or the EC population but importantly, that endothelial senescence alone can drive systemic metabolic dysfunction.

Age has been associated with significant changes in metabolism, leading to age-dependent increases in body weight, reduced insulin sensitivity and changes in lipid metabolism (89). Indeed, CVD is the leading cause of morbidity and mortality in obese individuals. White adipose tissue (WAT) is an organ responsible for regulating systemic energy homeostasis and is composed of visceral and s.c. WAT. Disruption of adipose tissue function has been linked to a chronic inflammatory state that can deregulate vascular homeostasis (90). Precursor cells required for maintaining adipocyte turnover and normal WAT function have been found to be senescent in obese human individuals and mice (91, 92). Further, studies on ECs isolated from adipose tissue biopsies from obese subjects showed that the visceral region had more inflammation and significantly higher senescent ECs compared to leaner subjects (93, 94). The ECs can be induced into senescence by conditioned medium from visceral adipose tissue from obese but not normal individuals, showing the potential paracrine effects of SASP (93). Mouse models have been used to dissect the role of obesity on the adipocyte and EC compartments. In endothelial-specific progeroid mice, the ECs were induced into early senescence. These mice showed metabolic impairment through adipose tissue dysfunction, specifically in s.c. inguinal WAT (95). The senescent endothelial-derived SASP (mainly IL-1a), induced senescent-like features in mature adipocytes but not on pre-adipocytes, indicating heighten the risk of senescent adipocytes with age (95). In normal mice on a high-calorie diet, p53/p21-driven EC senescence was observed in the aorta, skeletal muscle and lung. In endothelial-specific p53-deficient mice on a high-calorie diet, there was improved insulin sensitivity and reduced fat deposition (96). In contrast, overexpression of p53 in endothelium caused metabolic abnormalities in mice. Thus, endothelial senescence alone can drive systemic metabolic dysfunction thus providing a functional link between aging, the vasculature and metabolic disease.

### Diabetes

Type II diabetes mellitus (T2DM), also known as adult-onset diabetes, is a progressive condition in which the body becomes resistant to normal insulin function and/or gradual loss of insulin production in the pancreas. The reduction of insulin sensitivity is associated with a decrease in mass and function of insulin-producing pancreatic β–cells, leading to gradual high blood glucose or hyperglycemia. Many studies have shown that obesity and T2DM are closely linked (97), although diabetes can also occur in patients that are not obese and obese patients may not become diabetic. Aging is a known risk factor for both of these metabolic diseases, thus, suggesting that cellular senescence may provide a nexus between aging and metabolic disorders. Indeed β–cell senescence has been identified in pancreatic islets isolated from aged male mice, human donors with T2DM or with high BMI (98).

Vascular dysfunction has been widely reported in diabetic patients (99) and animal models (100). Of these diabetic retinopathy (DR) is a well-known microvascular complication of T2DM. Vision impairment in patients with DR is caused by macular edema and neovascularization. *In vitro* studies have shown that hyperglycemia can induce EC senescence (53, 101). Further, senescent ECs are found in aortic and retinal regions in rodent models of diabetes (102, 103). P53 activation is seen in the vasculature in both diabetic human and animal studies (101, 102, 103) and is associated with activation by hyperglycemic-induced production of advanced glycation end products (AGEs) and oxidative stress pathways (104). AGEs have been shown to induce EC dysfunction through p38/MAPK and ERK1/2 signaling pathways (105), which are associated with senescence and cell cycle regulation, respectively. One of the common secondary symptoms of T2DM is chronic non-healing wound. Diabetic patients are at risk of developing diabetic foot ulcers, a type of chronic non-healing wound, which can lead to lower limb amputation and death. Vascular dysfunction in diabetic patients has been associated with local tissue hypoxia and lower limb neuropathy resulting in poor healing of wounds (see review by Chao and Cheing (106)). Campisi *et al*. have demonstrated the need for fibroblasts and EC senescence in normal wound healing at least in animal models (43). Normal would healing is divided into four stages: (a) hemostasis, (b) inflammation, (c) proliferation and (d) remodeling. Fibroblasts and ECs are known to proliferate during physiological wound healing and senescence of these cells is required at the remodeling stages. However, in diabetic wounds, EC proliferation or neo-angiogenesis is compromised (107), resulting in impairment of vascular circulation to the wound. Consequently, the wound becomes hypoxic, leading to upregulation of p53, the upstream regulator of p21-dependent senescence. In diabetic murine wounds the senescence is observed mostly in macrophages (108) and the SASP from the accumulated senescent macrophages promotes fibroblast senescence and fibrosis. Topical silencing of p53 can increase EC numbers and improve diabetic wound healing (109). Thus, the difference between normal wound healing and diabetic wound healing would suggest that cell-type-specific senescence is important likely through the release of cell-type-specific SASP.

The EC compartment in diabetes is also compromised. Senescent ECs have been associated with inflammation and impaired angiogenesis in diabetic rats (110) and the endothelial progenitor cells (EPCs) derived from bone marrow are reduced and dysfunctional in diabetic patients (111). EPCs are known for maintaining vascular homeostasis and compensatory angiogenesis, which are also critical for wound healing. Thus, although senescent ECs exert a beneficial effect on normal wound healing, in pathological wound repair they appear to be detrimental.

### Cancer

Age is a risk factor for cancer development. The vasculature in solid tumors is both functionally and structurally abnormal, with chronic uncontrolled angiogenesis. In line with this proliferative phenotype, senescent ECs have been identified in human glioma likely activated to curb the proliferative response (112). However, this is also at the expense of promoting a proinflammatory milieu with increased Notch seen in human carcinomas, melanoma and human colorectal carcinomas (113). Endothelial Notch1 activity is linked to metastasis by promoting senescent, proinflammatory endothelium (114). In contrast, endothelial Notch3 activity was shown to limit tumor growth through apoptosis (115). In mice, ECs had a high expression of p16 in the primary tumor and metastatic sites in the lungs with constitutive active Notch 1. The senescent ECs were inflammatory, expressing high levels of the adhesion molecule VCAM1 that is associated with increased neutrophil infiltration into the tumor, a cell type associated with poor prognosis (114). The senescent cancer cells may also either induce angiogenesis or induce the ECs to undergo senescence through SASP. Thus, the EC senescence is both beneficial (inhibition of angiogenesis) but also detrimental (proinflammatory, facilitating neutrophil infiltration), which is consistent with the understanding of senescence in tumors, to act as a double-edged sword (29).

### Dementia

Clinical-pathology studies have suggested an overlap in cognitive impairment found in clinical Alzheimer’s disease (AD), with vascular pathologies (e.g. microinfarcts and microbleeds) (116). Comorbidity studies have found that patients with vascular dementia have higher prevalence of CVDs such as diabetes, atherosclerosis, coronary artery diseases and cardiac arrhythmia (117).

The major hallmarks of AD, the most common form of dementia, include the accumulation of aggregated extracellular amyloid and the intracellular-accumulation of neurofibrillary tangles, which can lead to neurodegeneration. AD can be characterized as familial or hereditary but the majority of AD cases are age-related.

The blood-brain barrier (BBB), which is part of the neurovascular unit, consists of ECs, pericytes, and astrocytes and regulates solute passage between the blood and the brain. BBB breakdown occurs early, even before deposition of amyloid-beta and cognitive decline (118, 119) suggesting an intact BBB is essential. The concept that endothelial dysfunction is a critical link in the development of AD, is gaining strength. Reduction in the cerebral blood flow, impaired hemodynamic response and vascular reactivity have been detected in patients in the early stages of AD and across people with normal aging-to-mild cognitive impairment (120). The increased deposition of amyloid plaque in brain microvessels, termed cerebral amyloid angiopathy is associated with microbleeds and cerebral hemorrhages (121). However, drug trials involving the use of anti-amyloid have shown that although it is effective in reducing plaque burden in AD patients, it did not reverse neurovascular dysfunction or improve cognitive function (122).

Senescence of microglia, astrocytes, pericytes, oligodendrocytes, oligodendrocyte progenitor cells, and neurons (123, 124, 125, 126, 127, 128) have been found in the brain and this senescence has been attributed to the development of AD and Parkinson’s disease. Although senescent ECs have not been identified in the brain of patients with these neurodegenerative diseases, many *in vitro* studies have shown that oligomeric β-amyloid can induce SIPS in ECs (54, 129).

Although a definitive understanding of EC senescence and AD development is still lacking, evidence is accumulating of its potential importance. A number of senescence-associated markers are seen in the endothelium in the brain. For example, a recent population-based study has shown positive correlation between DNA damage and p53 in the endothelium in human brain samples of AD (130). Further, the authors concluded that endothelial DNA damage and senescence is associated with aging and it may occur independently of AD pathology. A recent single-cell RNASeq study by Kiss *et al.* has detected a higher prevalence of senescent brain ECs in aged mice (51). These senescent brain ECs expressed a unique SASP profile that was different from senescent microglia, astrocytes and oligodendrocytes. Senescent ECs expressed increased levels of *Kitl*, *Plat*, *Igfbp7*, *Cxcl12*, and *Ctnnb1*. *Cxcl12* and *Ctnnb1* are related to leukocyte transendothelial migration, suggesting a mechanism for increased inflammation. Tissue plasminogen activator (TPA or Plat), is essential for clot lysis on the endothelial cell surface (131) and Ctnnb1 (β-catenin) is involved in cell–cell adhesion (132). Studies from progeroid mice have shown that senescent EC have poor tight junction organization (127) and tight junction proteins claudin-5 and occludin are found to decrease in expression in AD mice (133). A further example is Notch1 where studies have suggested it maybe a potential biomarker for AD (134, 135). Interestingly, the gamma secretase enzyme, involved in the cleavage of Notch receptor, is one of two enzymes primarily involved in the processing of the amyloid precursor protein into neurotoxic β-amyloid peptides. *In vitro* Notch1 expression was shown to be increased by β-amyloid treatment in brain ECs (134) and Notch1 activation induces EC senescence (85). Further, activated Notch1 is associated with vascular pathology in AD. Chronic hippocampal expression of cleaved Notch intracellular domain NICD was found to promote vascular thickening and amyloid deposition in a rat model of early AD and chronic activation of Notch1 led to a decreased cerebral blood flow in early AD in a transgenic rat model (136). Thus, identification of senescent ECs in AD, and their role in the BBB awaits further investigation.

## Senotherapeutics

The damaging role of senescent cells in disease development has really been cemented through the use of transgenic mice (e.g. INK-ATTAC, p16-TMR) that can selectively ablate senescent cells (137). With loss of senescent cells, these mice showed a reduced incidence of age-related pathologies in kidney, heart and brain.

The field of senolytics, drugs that selectively eliminate senescent cell, is gaining momentum. Dasatinib and Quercetin (D+Q) were some of the first senolytics, which remove senescent cells* in vitro* and in progeroid mice through targeting of the anti-apoptotic pathways in senescence (138). Long term oral treatment of D+Q have been shown to improve vascular function in aged or atherosclerotic mice at a level similar to genetic clearance of senescent cells in aged INK-ATTAC mice (139). The D+Q combination has been shown to efficiently reduce senescence cell burden in phase I trials for several senescence-related diseases such as diabetic kidney disease (NCT02848131) (140). Further, the D+Q combination have also been shown to alleviate physical dysfunction seen in senescence-associated disease such as idiopathic pulmonary fibrosis (NCT02874989) (141), however, it remains unclear whether senescent cells burden was reduced.

Recently, two further senolytics have been trialed. Navitoclax, which targets the Bcl-2 family of anti-apoptotic factors (142) and Venclexta (a small molecule BH3 mimetic) which blocks diabetes by targeting senescence (143). Further studies have shown that D+Q exhibit cell and pathway selectivity (140), thus demonstrating the need for expansion of our senolytic repertoire to provide cell- and disease-specific drugs for future use. A recent study has shown that sustained ablation of p16 senescent liver sinusoidal ECs promotes liver and perivascular fibrosis (144) which can be detrimental to healthspan. Thus, selective and time-dependent removal of the senescent population of cells is likely to influence the success of senolytics in the clinic. In contrast to senolytics that target the anti-apoptotic pathway, a novel study has demonstrated a drug called SSK1, which specifically eliminates senescent cells through the activity of lysosomal β-galactosidase with more superior efficacy than common senolytics (145). Other types of senescence targeting drugs with different specificities are being developed. These include senomorphics, drugs that modulate the inflammatory SASP profile without killing senescent cells. Studies using senolytics and senomorphics on disease/senescent endothelium have been well-reviewed by Kim and Kim (146). Finally, senescence immunotherapy provides another therapeutic strategy. At present, it is being used for treatment of cancers but it is emerging as a promising alternative to senolytics to clear senescent cells under the notion that different immune cells are capable of selectively identifying and removing unique senescent cells. Current senescent immunotherapy findings can be found in reviews by Kim and Kim (146) and Song *et al*. (147).

## Summary and future directions

ECs play a critical role in vascular homeostasis, regulation of inflammation and thrombosis and maintenance of organ function. This review outlined our existing understanding of senescent ECs and their contribution to cardiovascular disease, metabolic disease and dementia. Although senescence was initially considered as an all-encompassing phenotypic change, it is now apparent that each cell type exhibits an unique and distinguishing senescence phenotype, one that may also be tissue specific. Hence our understanding of endothelial senescence is still in its infancy. Current findings have indicated that specific depletion of senescent cells reverses age-related changes and prolongs life span. However, caution should be urged as cellular senescence also plays important physiological roles such as in tissue development, wound healing and tumor inhibition. To achieve optimal success in targeting senescence it will be imperative to have a thorough knowledge of the senescent cell type at play in disease, and their spatiotemporal expression in order to deliver the most appropriate senolytic, senomorphic or drug combination.

## Declaration of interest

Jennifer Gamble is a Senior Editor of *Vascular Biology*. Jennifer Gamble was not involved in the review or editorial process for this paper, on which she is listed as an author.

## Funding

The authors are supported by the National Health and Medical Research Council of Australia Ideas Grant (#GNT1183057).

## Author contribution statement

K K T, P C, Y Z, M A V and J R G contributed to writing this review.
